# Proposed requirements for hypoallergenic formulae to be used in the treatment and prevention of cow's milk allergy

**DOI:** 10.1186/2045-7022-1-S1-S72

**Published:** 2011-08-12

**Authors:** Antonella Muraro

**Affiliations:** 1 Padua General University Hospital, University of Padua, Food Allergy Referral Centre, Department of Pediatrics, Padua, Italy

## 

There are a large number of commercially available milk formulae labelled as "hypoallergenic". However, only a minority of these comply with the criteria established in the guidelines of Subcommittee on Nutrition and Allergic Disease of the American Academy of Pediatrics.

As far as the treatment of cow's milk allergy is concerned, the extensive hydrolysed protein formulae and aminoacid-based formulae are the only two preparations that meet the standards required for hypoallergenicity, defined as absence of reactions in 90% allergic patients with 95% confidence. However, even in these cases there is great variability in the content of the extensive hydrolysed formulas on the market and, for some of them, the clinical data in support of the claim of hypoallergenicity are missing. In addition, other products known as "partially hydrolysed formulae" have previously been advertised as being safe for cow’s milk allergic patients but turned out to be inadequate and responsible for anaphylactic reactions in many cases. These data underline the fact that it is mandatory to define the criteria in terms of peptidic content and preclinical profile of any formula put on the market as hypoallergenic formula for treatment of cow's milk allergy.

In the case of prevention of cow’s milk allergy, the data currently available are incomplete since no study has yet been published that meets all the criteria recommended by the American Academy of Pediatrics. Nonetheless, the studies conducted to date seem to indicate a greater efficacy of extensive hydrolysed protein formulae over partially hydrolysed formulae, although the latter may present nutritional advantages and lower cost.

In conclusion, further efforts are required in the characterisation of the commercially available milk formulae used for treatment and prevention of cow’s milk allergy. In the absence of well-documented studies proving the prophylactic value of partially hydrolysed formulae, children at high risk of atopy should be fed with a prophylactic hypoallergenic diet based on extensive hydrolysed formulas.

